# Homology of neocortical areas in rats and primates based on cortical type analysis: an update of the Hypothesis on the Dual Origin of the Neocortex

**DOI:** 10.1007/s00429-022-02548-0

**Published:** 2022-08-12

**Authors:** Miguel Ángel García-Cabezas, Julia Liao Hacker, Basilis Zikopoulos

**Affiliations:** 1 Department of Anatomy, Histology and Neuroscience, School of Medicine, Universidad Autónoma de Madrid, Madrid, Spain; 2 Neural Systems Laboratory, Department of Health Sciences, Boston University, Boston, MA USA; 3 Human Systems Neuroscience Laboratory, Department of Health Sciences, Boston University, 635 Commonwealth Ave., Room 401D, Boston, MA 02215 USA; 4 Department of Anatomy and Neurobiology, Boston University School of Medicine, Boston, MA USA; 5 Graduate Program in Neuroscience, Boston University, Boston, MA USA; 6 Present Address: Department of Neurology, The Children’s Hospital of Philadelphia, Philadelphia, PA USA

**Keywords:** Cortical area, Cortical layer, Cortical type, Cytoarchitecture, Homology, Phylogeny

## Abstract

Sixty years ago, Friedrich Sanides traced the origin of the tangential expansion of the primate neocortex to two ancestral anlagen in the allocortex of reptiles and mammals, and proposed the Hypothesis on the Dual Origin of the Neocortex. According to Sanides, paraolfactory and parahippocampal gradients of laminar elaboration expanded in evolution by addition of successive concentric rings of gradually different cortical types inside the allocortical ring. Rodents had fewer rings and primates had more rings in the inner part of the cortex. In the present article, we perform cortical type analysis of the neocortex of adult rats, Rhesus macaques, and humans to propose hypotheses on homology of cortical areas applying the principles of the Hypothesis on the Dual Origin of the Neocortex. We show that areas in the outer rings of the neocortex have comparable laminar elaboration in rats and primates, while most 6-layer eulaminate areas in the innermost rings of primate neocortex lack homologous counterparts in rats. We also represent the topological distribution of cortical types in simplified flat maps of the cerebral cortex of monotremes, rats, and primates. Finally, we propose an elaboration of the Hypothesis on the Dual Origin of the Neocortex in the context of modern studies of pallial patterning that integrates the specification of pallial sectors in development of vertebrate embryos. The updated version of the hypothesis of Sanides provides explanation for the emergence of cortical hierarchies in mammals and will guide future research in the phylogenetic origin of neocortical areas.

## Introduction

The main structural feature of the cerebral cortex is the arrangement of cortical neurons and glial cells in layers that run parallel to the surface of the brain. These layers are not homogeneous from one cortical area to another but vary systematically in gradients of laminar elaboration that have been traced across the cortical quilt of all the species of mammals examined so far. In marsupials and monotremes, cortical gradients of laminar elaboration expand from allocortical areas (primary olfactory and hippocampal areas) of 2–3 layers to periallocortical and proisocortical areas of 5 layers that lack or have rudimentary granular layer IV (Abbie 1940, 1942). In rodents, cortical gradients further expand through proisocortical areas into isocortical (eulaminate) areas that have 6 well-developed layers, including fully developed granular layer IV (Sanides 1970; Goulas et al. 2018; García-Cabezas et al. 2019). In primates, the isocortex expands significantly with the addition of more areas of 6 layers; these eulaminate areas show progressively more complex laminar differentiation, culminating in primary sensory (visual, auditory, and somesthetic) areas, which are the cortical areas with the finest laminar elaboration and thickest granular layer IV referred to as koniocortical areas (Sanides 1970; García-Cabezas et al. 2019). From a practical point of view, several cortical types of progressively more complex laminar elaboration can be operationally defined across gradients spanning from allocortical to koniocortical areas (Barbas 1986; García-Cabezas et al. 2020; John et al. 2021).

The progressive expansion of cortical gradients of laminar elaboration observed from marsupials and monotremes to primates led Friedrich Sanides to propose the Hypothesis on the Dual Origin of the Neocortex (Sanides 1962, 1970). Sanides developed this hypothesis based on his observations of the cortex of humans, non-human primates, cats, and rats and on the works of Raymon Dart (1934) on the cortex of reptiles and Andrew A. Abbie (1940, 1942) on the cortex of marsupials and monotremes [see Table 2 in García-Cabezas et al. (2019)]. According to the original enunciation of the Hypothesis on the Dual Origin of the Neocortex, the tangential expansion of the cerebral cortex is traced to two ancestral anlagen in the allocortex: the ancestral olfactory cortex and the ancestral hippocampal cortex, which form a continuous ring at the limit of each brain hemisphere. Thus, paraolfactory and parahippocampal gradients of neocortex emerged in evolution from this dual origin and developed parallel gradients of laminar elaboration that expanded from marsupial and monotremes to primates through successive concentric rings inside the allocortical (olfactory and hippocampal) ring. The most ancestral rings of agranular (absence of layer IV) and dysgranular (rudimentary layer IV) type neocortex are identified in all mammals (agranular neocortex is periallocortical and dysgranular neocortex is proisocortical). In contrast, the last stage of cortical differentiation that emerged in evolution, which includes koniocortical areas (primary visual, somesthetic, and auditory areas), is identified in primates surrounded by all the other rings.

Therefore, cortical types of progressively more complex laminar elaboration are arranged topographically (in each species examined) and topologically (across species) in concentric rings, the outer rings being the oldest in evolution and the inner being the newest (Sanides 1962, 1970). The oldest rings (allocortex; agranular and dysgranular neocortex) are located in the adult brain of primates at the limit of the hemisphere and are collectively called limbic areas [see Fig. 1 in Barbas (2015)].

Modern developmental data support several aspects of the Hypothesis on the Dual Origin of the Neocortex. For instance, studies of neurogenesis in non-human primates [see Fig. 2 in Rakic (2002)] showed that neocortical areas of simpler laminar elaboration, like limbic area 24, finalize their development earlier than areas of progressively more complex laminar elaboration, like the primary visual area. In human embryos, cellular proliferation in prospective neocortical areas of simpler laminar elaboration is lower compared to prospective areas of more complex laminar elaboration, which have higher proliferative activity in the outer subventricular zone [OSVZ; Reillo et al. (2011)]. Also, primate specializations of the developing cortex, like the OSVZ (Smart et al. 2002; Dehay et al. 2015), are absent in prospective areas of simpler laminar elaboration and appear and progressively increase in thickness and cellularity in prospective areas of more complex laminar elaboration (Barbas and García-Cabezas 2016). Thus, the developmental architecture of prospective neocortical areas of simple laminar elaboration (limbic areas) in humans resembles overall the developmental architecture of the rat neocortex (Barbas and García-Cabezas 2016; García-Cabezas et al. 2019), supporting more recent phylogenetic origin of neocortical areas of more complex laminar elaboration (eulaminate) in primates, as proposed by Sanides (1970).

Modern studies on causal mechanisms of pallial patterning in vertebrate embryos have identified embryonic organizers, like the hem, the septocommissural plate, and the anti-hem, that provide partial explanation of the specification and patterning of concentric pallial sectors during early development of vertebrates (Medina and Abellan 2009; Subramanian et al. 2009; Puelles et al. 2019). But patterning studies of the cortex also point at some statements of the Hypothesis on the Dual Origin of the Neocortex that seem to be inaccurate as enunciated by Sanides. Chiefly, genoarchitectonic studies in vertebrate embryos have defined four pallial sectors (medial, dorsal, lateral, and ventral pallia) that are present in fish, amphibia, reptiles, and mammals. In vertebrate species, the hippocampal formation develops from the medial pallium; the primary olfactory cortex develops from the ventral pallium; and most of the neocortex develops from the dorsal pallium with contributions from the lateral pallium (Medina et al. 2011; Puelles 2017; Suryanarayana et al. 2017; Pessoa et al. 2019; Puelles et al. 2019). The presence of four pallial sectors, including the dorsal pallium, in fish, amphibia, reptiles, and mammals should be taken into account to update the original enunciation of the Hypothesis on the Dual Origin of the Neocortex: it seems that the rings of mammalian neocortex did not appear de novo in mammals, but expanded, likely under the direction of the hem, the septocommissural plate, the anti-hem, and other organizers not yet identified, within two pallial domains (dorsal and lateral pallia) that were likely present in the common ancestor of fish and land vertebrates.

An interesting application of the Hypothesis on the Dual Origin of the Neocortex in comparative neurobiology is proposing homologies of cortical areas across mammalian species. If two given cortical areas in two different species of mammals are in the same ring of neocortex, they could be homologous. In contrast, if two cortical areas (or cortical sectors) in two species are in different rings of neocortex they are unlikely to be homologous (Sanides 1970). According to most definitions of homology, two body characters (like parts of the cerebral cortex) in two species are homologous if they originated in the same organ of a common ancestor species (Wiley and Lieberman 2011; Wagner 2014). Such statements about homology of organs across species are indemonstrable, but researchers can find criteria to propose substantiated homology hypotheses. For instance, the topological position of a given body character in relation to body plans and the causal mechanisms in development of that character should be comparable among the species of interest for advancing homology hypotheses (Wagner 1989; Puelles and Medina 2002; Nieuwenhuys and Puelles 2016). Therefore, homology hypotheses of cortical areas or parts of the cerebral cortex among mammalian species based on the Hypothesis on the Dual Origin of the Neocortex should take into account (1) the topological distribution of cortical types (rings) studied in Nissl-stained sections of adult brains of the two species of interest, and (2) the location of the areas or parts of the cortex tested for homology across cortical types in Nissl-stained sections of adult brains of the two species of interest. Such hypotheses should not contradict causal mechanisms of pallial patterning studied in vertebrate (chiefly mammalian) embryos.

In the present article, we perform cortical type analysis of the neocortex of adult rats, Rhesus macaques, and humans to propose hypotheses on homology of cortical areas across these species within the theoretical framework of the Hypothesis on the Dual Origin of the Neocortex of Sanides. Such hypotheses on homology are relevant for understanding the phylogeny of the human cerebral cortex, but also to extrapolate experimental data obtained from animal models (e.g., tract tracing studies of cortical connections) to humans. We use Nissl-stained sections of rat brains and the micrographs of Nissl-stained sections of the Rhesus macaque cortex published in *The Neocortex of Macaca mulatta* by von Bonin and Bailey (1947). We also perform the same analysis in Nissl-stained sections of the human ventromedial prefrontal cortex (VMPFC), a region in which gradients of laminar elaboration span from allocortical precommissural hippocampus to eulaminate areas in the frontal pole (Mackey and Petrides 2014; García-Cabezas et al. 2020), to provide direct comparison of cortical gradients of laminar elaboration between primate (human) and rat species. We provide tables with the names and abbreviations of cortical areas across the neocortex of rats and Rhesus macaques with their corresponding cortical type. We compare the elaboration of cortical types across rats and primates and show their topological distribution in simplified flat maps of the cerebral cortex of these species. We show that neocortical areas of the outer rings of cortex have comparable laminar elaboration in rats and primates, while most 6-layer eulaminate areas in the innermost rings of primate neocortex do not seem to have homologues with the equivalents in the rat counterparts. Finally, we address the strengths and potential shortcomings of the Hypothesis on the Dual Origin of the Neocortex in the context of modern studies of pallial patterning to propose an updated version of this hypothesis and suggest experiments to help guide future research in the phylogenetic origin of neocortical areas.

## Materials and methods

### Rat and human brains

We analyzed the laminar structure of the cerebral cortex in Nissl-stained sections of adult albino (Sprague-Dawley) rats (*n* = 2) that were generously provided by Dr. Alan Peters. Albino rats were originally obtained from the Charles River Breeding Laboratories [Wilmington, MA; see Feldman and Peters (1978)]. Human brains (*n* = 4; data summarized in Table 1) were obtained from the National Disease Research Interchange (NDRI), and Anatomy Gifts Registry. The study was approved by the Institutional Review Board of Boston University.

**Table 1:** Data of human subjects

Human subjects	Sex	Age (years)	Internal Cat. #
MD12112758	F	58	HAW
VA12103176	F	67	HAY
ND14162	M	55	HCP
ND11109	M	38	HCD

### Tissue processing and Nissl staining

The cases offered by Dr. Alan Peters were anesthetized with 0.5 ml of 36% chloral hydrate, perfused intracardially with saline followed by 10% formalin, and postfixed in the same fixative for 2 weeks. Brains were removed from the skull, embedded in paraffin, and cut in 15 µm thick coronal sections that were stained with cresyl violet (Nissl) and fast blue (myelin) as described (Feldman and Peters 1978).

Donated post-mortem human brains were fixed by immersion in 10% formalin. Brain peduncles and the corpus callosum were cut upon arrival in the Human Systems Neuroscience Laboratory at Boston University. The surfaces of each hemisphere were photographed with a digital camera (Canon EOS 5N) and each hemisphere was sliced in 1-cm-thick coronal slabs. Brain slabs were photographed (anterior and posterior surfaces) and postfixed in 10% formalin for 2–4 days. After post-fixation, smaller blocks of the slabs containing the VMPFC were cut, photographed, cryoprotected in a series of sucrose solutions (10–30% in 0.01 M PBS), and frozen in 75 ºC isopentane (Thermo Fisher Scientific, Pittsburg, PA, United States) for rapid and uniform freezing (Rosene et al. 1986). Blocks were cut in the coronal plane at 50 µm on a freezing microtome to collect 10 consecutive series of sections. Some blocks were embedded in agar (6%) without cryoprotection and cut at 50 or 75 µm using a vibratome (PrecisionaryVF-700, Precisionary Instruments Inc., Greenville, NC, USA). A series of sections of each block was mounted in gelatin coated slides (Gelatin Type A, G8-500, Fischer Chemicals, Fair Lawn, NJ, USA) and processed for Nissl staining as described (García-Cabezas et al. 2016).

### Optical microscopy and photography of Nissl-stained sections

Nissl-stained sections through the entire rat cortex and through the human VMPFC were examined with an optical microscope (Olympus, BX51) at low (2x: PlanApo 2x/0.08 Japan; 4x: UPlanFl 4x/0.13 Japan) and medium (10x: UPlanFL 10x/0.30 Japan) magnification. For the rat cortex we followed the areal parcellation provided by the Atlas of Karl Zilles [*The Cortex of the Rat: A Stereotaxic Atlas* (1985)]. For the human VMPFC, a region in which gradients of laminar elaboration span from the allocortical precommissural hippocampus to eulaminate areas in the frontal pole (Mackey and Petrides 2014), we followed our previous description (García-Cabezas et al. 2020).

Micrographs (Tiff files) of representative columns of each neocortical area encompassing all the layers were taken at 10 × with a CCD camera (Olympus DP70). We assigned a number to each micrograph of cortical areas of the rat and kept a list of each number and the corresponding area. Tiff files of each micrograph identified with the number were imported to PowerPoint software for blind cortical type analysis on a computer.

To assemble figures, we imported selected images into Adobe Illustrator CC software (Adobe Systems Inc., San José, CA, USA). Minor adjustment of overall contrast and brightness were made but the micrographs were not retouched.

### Scanning of micrographs of the Atlas of the Rhesus macaque cortex of von Bonin and Bailey (1947***)***

For cortical type analysis of the neocortex of the Rhesus macaque, we used the Atlas of von Bonin and Bailey (1947). We chose this atlas because is the only published work that charts the entire cortical surface of the Rhesus macaque through Nissl-stained sections and shows micrographs of the areas described. Von Bonin and Bailey took micrographs of Nissl-stained sections at 58 points across the surface of the cortical quilt of the Rhesus macaque (*Macaca mulatta*). They also marked in maps of the cortical surface the points in which micrographs were taken. These micrographs are shown in 58 plates labeled with Roman numerals, the symbol of the corresponding area according to the nomenclature of von Economo and Koskinas (1925/2008), and page numbers. We scanned the micrographs of neocortical areas with a scanner (Epson Perfection 1240U Photo, Seiko Epson Corp, Nagano-ken, Japan) skipping the identifier of area to allow for blind analysis. Tiff files of each scan were imported to PowerPoint software for cortical type analysis on a computer.

### Map elaboration

We used Adobe Illustrator CC software to redraw selected maps of the rat cortex by Zilles (1985) and of the Rhesus macaque cortex by von Bonin and Bailey (1947). We then represented cortical types in grayscale on these maps as we have done before for the human cortex (García-Cabezas et al. 2020).

We also represented the distribution of cortical types in simplified flat maps of rats and primates to help visualize their topological arrangement across these species. We also represent a map of cortical types in monotremes based on the studies of (Abbie 1940) because these animals are considered the most primitive of all extant mammals (Deakin et al. 2012).

## Results

### Topography and topology of parts of the cerebral cortex

Spatial relations between parts of the nervous system (or any other organ) can be described from two points of view. First, *topography* studies the distribution of parts on the surface of or within an organ; in the present article, we study the topographical distribution of cortical types across the cerebral cortex in adult brains of rats and primates. Second, *topology* studies the invariant neighborhood relations between parts on the surface of or within an organ unaffected by the continuous change of shape or growth of this organ; in the present article, we study the topological distribution of cortical types (rings) unaffected by the expansion of the cerebral cortex across phylogeny [for definitions of topography and topology applied to brain structures see Nieuwenhuys (2011, 2017)].

**Fig. 1:**
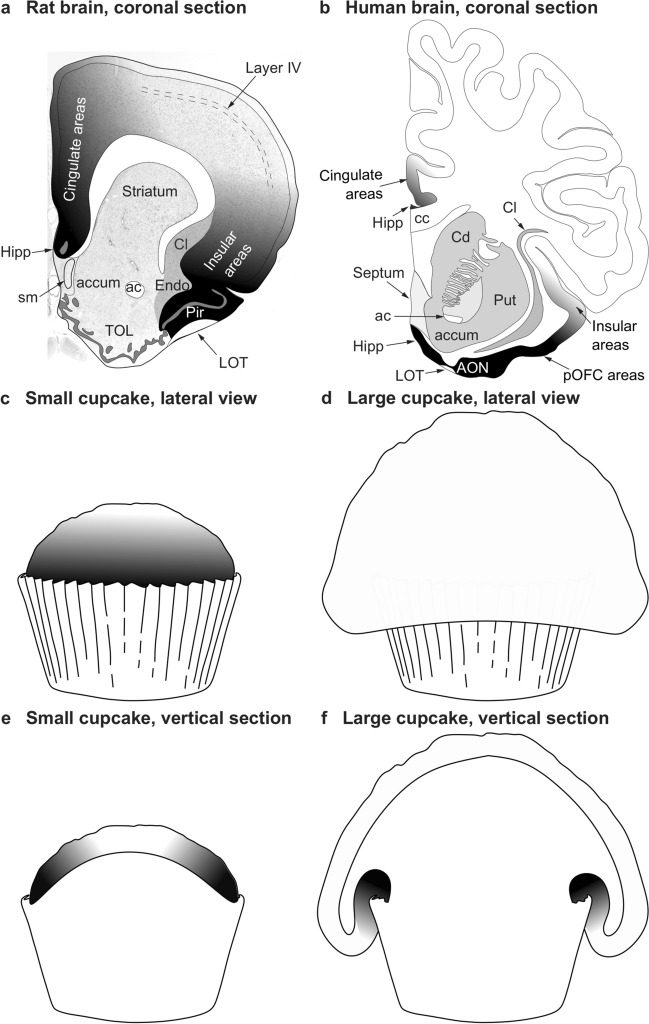
Topography and topology of cortical types. **a** Coronal section of the rat brain through the olfactory tubercule. Dorsolateral isocortical (eulaminate with well-developed layer IV, white and light shades of gray) areas are flanked on the medial side by the allocortical precommissural hippocampus (Hipp, black) and cingulate agranular and dysgranular areas (dark shades of gray); Isocortical areas are flanked on the ventrolateral side by allocortical olfactory areas (Pir, black) and insular agranular and dysgranular areas (dark shades of gray). **b** Coronal section of the human brain through the nucleus accumbens. Dorsolateral isocortical (eulaminate with well-developed layer IV, white) areas are flanked on the ventromedial side by the allocortical precommissural hippocampus (Hipp, black) and cingulate agranular and dysgranular areas (dark shades of gray); isocortical areas are flanked on the ventral side by allocortical olfactory areas (AON, black) and insular and posterior orbitofrontal (pOFC) agranular and dysgranular areas (dark shades of gray). **c** Lateral view of a small cupcake. **d** Lateral view of a large cupcake. **e**, Vertical section of the small cupcake in c; the gray scale in c and e resembles the distribution of cortical types on the rat brain in **a**. **f** Vertical section of the large cupcake in d; the gray scale in **d** and **f** resembles the distribution of cortical types on the human brain in **b**. The topological neighborhood relations of cortical types (shades of gray) in **a**, **c**, and **e** are preserved in **b**, **d**, and **f**, despite the extensive expansion of isocortical areas in primates that expand over limbic areas like cupcake batter flows over a cupcake mold. *ac* anterior commissure, *accum* nucleus accumbens, *AON* anterior olfactory nucleus in the primary olfactory cortex, *cc* corpus callosum, *Cd* caudate nucleus, *Cl* claustrum, *Endo* endopiriform nucleus, *Hipp* anterior extension of the hippocampal formation, *LOT* lateral olfactory tract, *Pir* piriform cortex in the primary olfactory cortex, *pOFC* posterior orbitofrontal cortex, *Put* putamen, *sm* stria medullaris, *TOL* olfactory tubercule. **a**, **b** are modified from Fig. 3 in Garcia-Cabezas and Zikopoulos (2019)

The topographical arrangement of cortical types in concentric rings was identified by Sanides in Nissl and myelin-stained sections of adult brains. The outer location of allocortex, agranular neocortex (periallocortex) and dysgranular neocortex (proisocortex) rings is easy to spot in coronal sections of rodent brains that show dorsolateral isocortical areas flanked, on the medial side, by cingulate agranular and dysgranular areas and, on the ventrolateral side, by insular agranular and dysgranular areas (Fig. 1a). In primates, the topographical analysis of cortical types shows that agranular neocortex (periallocortex) and dysgranular neocortex (proisocortex) are partially or totally covered by isocortical areas (Fig. 1b). This is due to the extensive expansion of isocortical areas in primates that expand over limbic areas like cupcake batter flows over a cupcake mold (Fig. 1c–f). Thus, topographically, agranular and dysgranular areas may appear more “central” than “peripheral” in primates compared to rodents but, topologically, agranular and dysgranular limbic areas flank isocortical areas both in rodents and primates.

In this article we describe the topographic distribution of cortical types in rats, macaques, and humans. Then, we identify the invariant neighborhood relations (topological relations) of cortical types across rats, macaques, and humans.

### Terminology of cortical types

We first define the terms that will be used through the present article to label parts of the cerebral cortex in the three species analyzed (Table 2).

**Table 2:** Nomenclature of cortical types

Present article	Allocortex (Archicortex and paleocortex)	Mesocortex (Neocortex *non *sensu stricto)		Isocortex (Neocortex sensu stricto)					
		Agranular/Periallocortex	Dysgranular/Proisocortex	Eu I	Eu II		Eu III		Konio
Barbas (1986)	–	1	2	3	4		5		–
Barbas and Pandya (1989)	–	Pall	Pro	Eu I	Eu II		Eu III		–
Barbas and Rempel-Clower (1997)	–	Level 1	Level 2	Level 3	Level 4		Level 5		–
Hilgetag et al. (2016)	1		2	3	4	5	6	7	8
Joyce and Barbas (2018)	–	Agranular	Dysgranular	Eu I	Eu II		Eu III		–
Zhang et al. (2020)	–	Limbic		Eulaminate					Konio
García-Cabezas et al. (2020)	Allocortex	Agranular	Dysgranular	Eu I	Eu II		Eu III		Konio cortex
John et al. (2021)	–	Agranular	Dysgranular	Eu I	Eu II		Eu III		Konio

*Eu* Eulaminate, *Konio* Koniocortex

The cerebral cortex can be divided in areas and types according to its microscopic structure as seen in Nissl-stained sections. *Cortical areas* are parts of the cortex with characteristic cytoarchitectonic features (or a combination of features) that are absent in other parts. In contrast, *Cortical types* are defined based on “the constant variations that one observes in each of the layers in different regions” (von Economo and Koskinas 1925/2008; von Economo 1927/2009). For cortical type analysis, instead of looking for particular cytoarchitectonic features, the observer looks across the cortical quilt for gradual and systematic variation of laminar features, like number and prominence of layers, or size of largest neurons in layers, to name a few (García-Cabezas et al. 2020). Cortical types are topologically arranged in concentric rings across the surface of the cortex (Sanides 1970); thus, we will use *Cortical ring* as synonym of cortical type. *Pallial sectors* are parts of the cortex that are patterned during early stages of development due to the action of morphogenetic proteins; four pallial sectors (medial, dorsal, lateral, and ventral pallium) are defined using genoarchitectonic techniques (Puelles 2017).

Several cortical types can be defined across gradients of laminar elaboration. We will briefly describe the laminar features of cortical types and their topological relations across the gradient of laminar elaboration. *Allocortex* is the part of the cortex with the simplest laminar elaboration. The hippocampal formation (archicortex) and the primary olfactory cortex (paleocortex) are the 2 parts of the allocortex and have areas with 2 or 3 layers. In contrast to allocortical areas, the *Isocortex*, is composed of areas with 6 well-developed layers. We use *Eulaminate* area as synonym of isocortical (6-layer) area. We also use *Neocortex *sensu stricto as synonym of isocortex. The isocortex is not homogeneous and several eulaminate types of progressive laminar elaboration can be defined. At the end of the isocortical gradation in primates are *Koniocortical areas*, which are eulaminate areas with the most complex laminar elaboration.

Between allocortical and isocortical areas is the *Mesocortex*, which is composed of areas that have more layers than the allocortex, but fewer than the isocortex; thus, mesocortical areas either lack or have rudimentary granular layer IV. We use *Neocortex non *sensu stricto as synonym of Mesocortex. Mesocortical areas that lack layer IV are *Agranular* and those which have rudimentary layer IV are *Dysgranular*. Agranular areas are adjacent to allocortical areas and are also called *Periallocortical*. Dysgranular areas are between agranular areas and isocortical areas and are also called *Proisocortical*. *Limbic* areas are those at the limit of the hemisphere; thus, allocortical and mesocortical areas are limbic areas.

In the present article we analyze cortical types in isocortical (neocortex sensu stricto) and mesocortical (neocortex *non *sensu stricto) areas. We do not analyze allocortical areas [primary olfactory cortex: piriform cortex and anterior olfactory nucleus; hippocampal formation: dentate gyrus, fields in Ammon’s horn, subiculum, presubiculum, parasubiculum, taenia tecta, indusium griseum; Nieuwenhuys et al. (2008); Puelles et al. (2019)]. Recent genoarchitectonic data suggest that the entorhinal cortex, which traditionally has been considered periallocortical, is allocortex (Puelles et al. 2019); therefore, we also excluded the entorhinal cortex for cortical type analysis.

### Principles of cortical type analysis

We will briefly describe the general principles of cortical type analysis. For more details, the reader is referred to our previous article on the human cerebral cortex (García-Cabezas et al. 2020).

Laminar architecture varies systematically across the entire surface of the neocortex in gradients of laminar elaboration with increasing development of granular layer IV and sharper definition of the other layers. We have traced cortical gradients in primates (García-Cabezas and Barbas 2017; García-Cabezas et al. 2017, 2019, 2020; Zikopoulos et al. 2018) and have identified the most useful laminar features for cortical type analysis using Nissl-stained sections. These features, summarized in Table 3, include development of layer IV, prominence (denser cellularity and larger neuron bodies) of deep (V–VI) or superficial (II–III) layers, definition of sublayers (e.g., IIIa and IIIb), sharpness of boundaries between layers, and presence of largest pyramids in layers III and/or V (García-Cabezas et al. 2020).

**Table 3:** Laminar features of cortical types in the neocortex

Laminar features	Agranular	Dysgranular	Eulaminate I	Eulaminate II	Eulaminate III	Koniocortex
Layer IV	Absent	Thin, irregular, discontinuous	Thin, regular, continuous	Thick, regular, continuous	Thick, regular, continuous	Thickest
More prominent laminar group	Deep V–VI	Deep V–VI	Deep V–VI and superficial II–III equally prominent	Superficial II–III	Superficial II–III	Superficial II–III (denser with small neurons)
Largest pyramids	V	V	V and III	III	III (larger)	III
Layers V–VI	Indistinct V and VI	Indistinct V and VI	Distinct V and VI	Sublayers in V and VI	Sublayers in V and VI	Sublayers in V and VI
Layers I–II boundary	Irregular	Slightly irregular	Sharp	Sharp	Sharp	Sharp

A key aspect of cortical type analysis is that transitions from one type to another are gradual without abrupt jumps along the gradients of laminar elaboration. Another aspect to consider is that some cortical areas defined in classical studies [*e. g.*, Brodmann (1909/1999); von Economo and Koskinas (1925/2008); von Bonin and Bailey (1947)] will have just one cortical type, others may be in the middle of transitions from one type to another, and others may be large enough to span through several cortical rings.

### Equivalence of cortical types across studies

Finally, we will make some comments regarding the objectivity of cortical type analysis. It must be taken into account that progressive elaboration of laminar structure of the cerebral cortex across gradients is an objective fact revealed by microscopic examination of Nissl-stained sections of mammalian brains. In contrast, the definition of cortical types across gradients of laminar elaboration is subjective, although not arbitrary. We defined 1 allocortical type, 2 mesocortical types (agranular and dysgranular) and 4 isocortical types (eulaminate I, II, II, and koniocortex) in the human cortex (García-Cabezas et al. 2020), other researchers defined 5 types in prefrontal areas (Barbas and Pandya 1989; Barbas and Rempel-Clower 1997), 6 types across the entire neocortex (Barbas 1986; John et al. 2021), or defined 8 types in visual areas of the Rhesus macaque (Hilgetag et al. 2016); others defined 3 types in the human cortex (Zhang et al. 2020), but other researchers may define fewer or more types (e.g., 4 types: allocortex, mesocortex, eulaminate, and koniocortex).

In Table 2, we summarize cortical type equivalences across the most relevant parcellation studies of the macaque and human cortex. The authors of most of these studies performed cortical type analysis based on qualitative features of laminar architecture following the seminal scheme of Helen Barbas for the macaque neocortex (Barbas 1986). The major exception to this rule is the study by Hilgetag et al. (2016), in which cortical type definition is based on laminar features and neuron density because eulaminate types have higher neuron density than mesocortical types. Hilgetag et al. (2016) used unbiased stereological methods to quantify neuron density across visual areas of the macaque and sorted these areas into types according to their laminar features and neuron density. They found that area V1 differed by about three ordinal scales from the densest eulaminate III prefrontal area and, thus, extended the classification from five cortical types for prefrontal areas of Helen Barbas (Barbas and Pandya 1989; Barbas and Rempel-Clower 1997) to eight types for the cortical visual system: V1 was assigned to cortical type 8 and several unimodal visual areas were assigned to cortical type 7 (e.g., V2) and cortical type 6 (e.g., V4). Thus, cortical type 8 defined by Hilgetag et al. (2016) based on neuron density is the equivalent of our Koniocortical type, cortical types 6 and 7 are subsumed in our eulaminate III, and some of the areas assigned to cortical type 5 fall in our eulaminate II. Also, Hilgetag et al. (2016) included the entorhinal cortex (area 28), which is part of the allocortex (Puelles et al. 2019), in their cortical type 1.

In summary, subjective interobserver differences are not arbitrary as long as the topological order of types is preserved. That means one can move across the cortical surface from one type to the next type up or down the gradient with increments no higher than 1. The topological distribution of cortical types across gradients of laminar elaboration from allocortex to koniocortex allows to relate classification schemes of cortical types of different studies to each other.

### The rat neocortex is mostly mesocortical

We performed cortical type analysis on Nissl-stained sections across the entire cortical quilt in rats. For that purpose, we took micrographs across all layers of neocortical areas described by Zilles (1985) for analysis of laminar features summarized in Table 3.

**Fig. 2:**
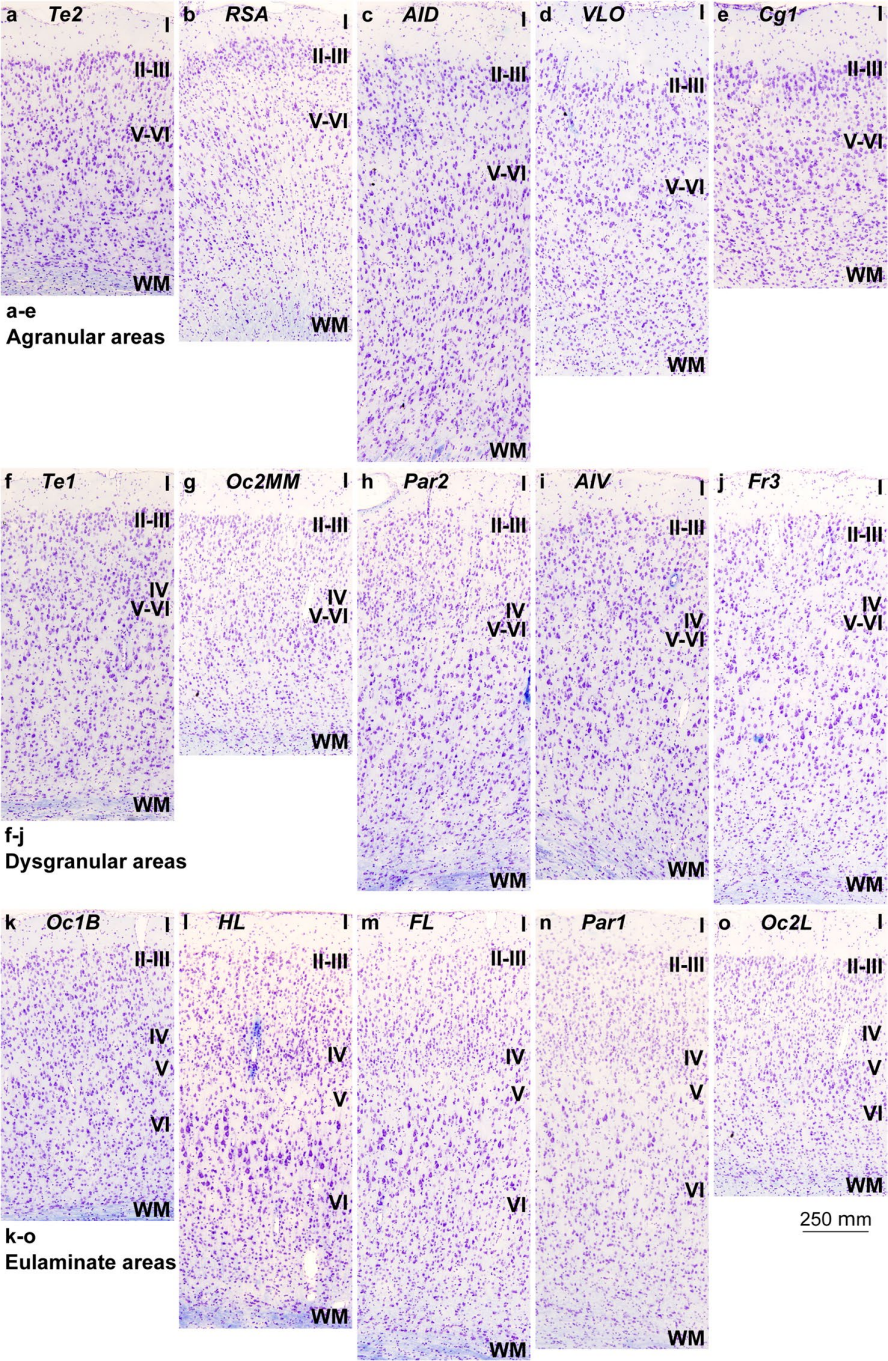
Cortical types across the rat cerebral neocortex. **a–e**, Micrographs of agranular mesocortical areas (Nissl staining). **f–j**, Micrographs of dysgranular mesocortical areas (Nissl staining). **k–o**, Micrographs of eulaminate areas (Nissl staining). Cortical areas are indicated according to Zilles (1985); see Table 4 for abbreviations of areas. Roman numerals indicate cortical layers. WM: white matter. Calibration bar in o applies to **a**–**o**

Mesocortical areas adjacent to hippocampal and olfactory allocortical areas showed overall simple lamination without layer IV, indistinct layers V–VI, and irregular boundary between layers I and II. These areas had more prominent and denser layers V–VI than layers II–III and the largest bodies of pyramidal neurons were in layer V (Fig. 2A–E). We categorized these areas as *Agranular*.

Other mesocortical areas adjacent to agranular areas also had simple lamination, but an incipient layer IV could be identified. In these areas, layers V–VI were more prominent than superficial layers II–III and the largest bodies of pyramidal neurons were in layer V (Fig. 2F–J). Layers V–VI were slightly more distinct than in agranular areas. We categorized these areas as *Dysgranular*.

Finally, few areas adjacent to dysgranular areas and apart from agranular areas had well-developed layer IV. In these areas, layers V–VI were still more prominent but superficial layers II–III were denser than in agranular and dysgranular areas. The largest pyramids, which in Hind Limb (HL) motor area were the largest bodies of pyramidal neurons across the entire cortex of the rat, were in layer V. Layers V–VI were well differentiated (Fig. 2K–O). These areas did not meet two criteria for eulaminate areas described in primates: equal prominence of superficial II–III and deep V–VI layers and largest pyramids equally distributed in layers III and V. In spite of this, they had overall the best laminar elaboration across the entire cortical quilt of the rat and had well-developed layer IV. We categorized these areas as *Eulaminate*.

**Fig. 3:**
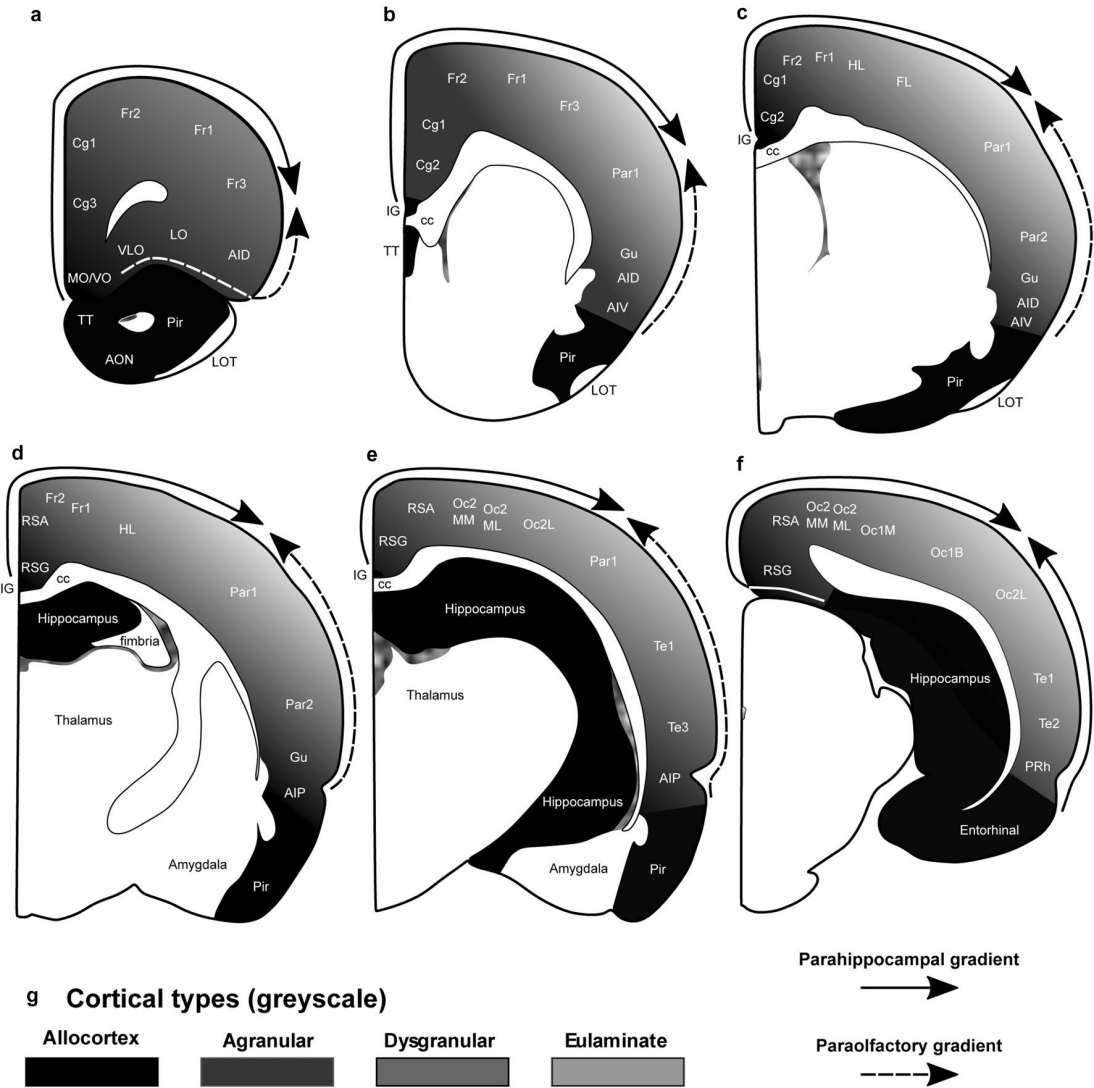
Distribution of cortical types across the rat cortex. **a–f**, Coronal maps of the rat brain according to Zilles (1985). **a** is the most rostral map and **f** is the most caudal map. Allocortical areas are colored in black; agranular mesocortical areas are colored with the darkest gray; dysgranular mesocortical and eulaminate areas are colored in progressively lighter grays. **g**, Grayscale of cortical types in **a**–**f**. Cortical areas are indicated according to Zilles (1985); see Table 4 for abbreviations of neocortical areas. *AON* anterior olfactory nucleus, *cc* corpus callosum, *IG* indusium griseum, *LOT* lateral olfactory tract, *Pir* piriform cortex, *TT* taenia tecta. Solid arrows indicate the parahippocampal gradient of laminar differentiation. Dashed arrows indicate the paraolfactory gradient of laminar differentiation

The areas of the rat neocortex according to Zilles (1985) and their corresponding cortical types are summarized in Table 4. We also show cortical types (in grayscale) and areas in coronal maps redrawn from Zilles (1985) (Fig. 3). Agranular, dysgranular, and eulaminate areas in the rat neocortex could be traced across 2 gradients (paraolfactory and parahippocampal) of laminar elaboration. The paraolfactory gradient (dashed arrow in Fig. 3A–E) started adjacent to the anterior olfactory nucleus and the piriform cortex and expanded in dorsal and medial direction. The parahippocampal gradient (solid arrow in Fig. 3A–F) started adjacent to the hippocampal formation; in rostral and middle levels, this gradient expanded from the taenia tecta and indusium griseum in dorsal and lateral direction (Fig. 3A–E). In caudal levels, this gradient expanded from hippocampal areas in dorsal and lateral direction and from entorhinal areas in dorsal and medial direction (Fig. 3F). Both paraolfactory and parahippocampal gradients converged in dorsally located eulaminate areas. Coronal maps in Fig. 3 show that most of the rat cerebral neocortex consists of agranular (52% of all areas) and dysgranular areas (32% of all areas); thus, most of the rat neocortex is mesocortical with fewer eulaminate areas, which constitute about 16% of all cortical areas.

**Table 4:** Areas in the cerebral cortex of the rat according to Zilles (1985) and their corresponding types

Abbreviation	Cortical area	Cortical type
AID	Agranular insular cortex, dorsal part	Ag
AIP	Agranular insular cortex, posterior part	Ag
AIV	Agranular insular cortex, ventral part	Dys
Cg1	Cingulate cortex, area 1	Ag
Cg2	Cingulate cortex, area 2	Ag
Cg3	Cingulate cortex, area 3	Ag
DPC	Dorsal peduncular cortex	Ag
FL	Forelimb area	Eu
Fr1	Frontal cortex, area 1 (primary motor cortex)	Dys
Fr2	Frontal cortex, area 2	Dys
Fr3	Frontal cortex, area 3	Dys
Gu	Gustatory cortex	Dys
HL	Hindlimb area	Eu
IL	Infralimbic area of the medial frontal cortex	Ag
LO	Lateral orbital area	Ag
MO	Medial orbital area	Ag
Oc1B	Occipital cortex, area 1 binocular part (primary visual cortex)	Eu
Oc1M	Occipital cortex, area 1 monocular part (primary visual cortex)	Dys
Oc2ML	Occipital cortex, area 2 mediolateral part	Dys
Oc2MM	Occipital cortex, area 2 mediomedial part	Dys
Oc2L	Occipital cortex, area 2 lateral part	Eu
Par1	Parietal cortex, area 1 (primary somatosensory cortex)	Eu
Par2	Parietal cortex, area 2 (supplementary somatosensory cortex)	Dys
PRh	Perirhinal cortex	Ag
RSA	Agranular retrosplenial cortex	Ag
RSG	Granular retrosplenial cortex	Ag
Te1	Temporal cortex, area 1 (primary auditory cortex)	Dys
Te2	Temporal cortex, area 2	Ag
Te3	Temporal cortex, area 3	Ag
VLO	Ventrolateral orbital area	Ag
VO	Ventral orbital area	Ag

### The Rhesus macaque neocortex is mostly isocortical

We performed cortical type analysis on the micrographs of Nissl-stained sections of the Atlas of the Rhesus macaque (*Macaca mulatta*) cortex of von Bonin and Bailey (1947). These authors adapted the terminology used by von Economo and Koskinas for areas in the human cortex (von Economo and Koskinas 1925/2008) to Rhesus macaque cortical areas. Accordingly, von Bonin and Bailey identified each cortical area of the Rhesus macaque with a symbol that comprises a Roman capital letter from the initial of the respective lobe (F for frontal, O for occipital, T for temporal, P for parietal, L for limbic cingulate, I for insular) and a calligraphic capital for the sequence of gyri within each lobe. The symbol of some areas also comprises a Greek subscript for the microscopic features of the area (Triarhou 2007).

von Bonin and Bailey (1947) surveyed 58 sites across the cortical quilt in Rhesus macaques and showed micrographs of each site in high quality micrographs. We applied the criteria summarized in Table 3 for cortical type analysis to the 58 micrographs and categorized the areas depicted as agranular, dysgranular, eulaminate I, eulaminate II, eulaminate III, and koniocortex. We will briefly describe the laminar features of each type.

Some areas had overall simple laminar differentiation, lacked layer IV, and had irregular boundary between layers I and II. Neurons in the deep layers (V–VI) of these areas were densely packed and superficial layers (II–III) were sparsely populated; the largest bodies of pyramidal neurons were in layer V. We categorized these areas as *Agranular*.

Other areas had rudimentary layer IV and their deep layers (V–VI) were slightly more prominent than superficial layers (II–III). The largest bodies of pyramidal neurons were in layer V and layers I and II were separated by a slightly irregular boundary. We categorized these areas as *Dysgranular*.

A third category of areas had thin but continuous and regular layer IV and deep (V–VI) and superficial (II–III) layers were equally prominent. The largest bodies of pyramidal neurons were equally distributed in layers III and V and the boundary between layers I and II was sharper than in agranular and dysgranular areas. Layers V and VI had sharper differentiation. We categorized these areas as *Eulaminate I*.

In other areas, layer IV was thicker than in Eulaminate I areas, superficial (II–III) layers were more prominent than deep (V–VI) layers, and the largest bodies of pyramidal neurons were in layer III. Layers V and VI had sharp differentiation and, in some areas, they were divided in sublayers. The boundary between layers I and II was also sharp. We categorized these areas as *Eulaminate II*.

Finally, some areas had laminar features comparable to Eulaminate II areas but with larger and more prominent pyramidal neurons in layer III. We categorized these areas as *Eulaminate III*. Only one koniocortical area, the primary visual area, was photographed by von Bonin and Bailey. This area had the thickest layer IV and superficial (II–III) layers were densely populated with small neurons. This was the only area photographed by von Bonin and Bailey that we categorized as *Koniocortex*.

**Fig. 4:**
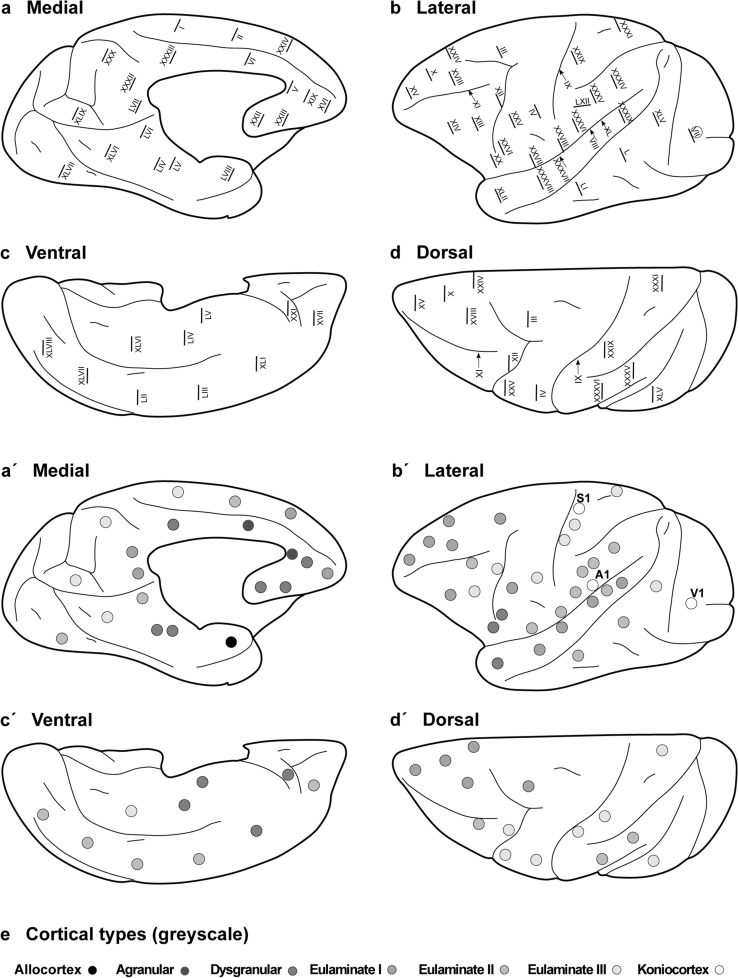
Distribution of cortical types across the Rhesus macaque cortex. Medial (**a**), lateral (**b**), ventral (**c**), and dorsal (**d**) views of the Rhesus macaque cerebral cortex; Roman numerals indicate the location of plates in the Atlas of von Bonin and Bailey (1947). **a´–d´**, Medial, lateral, ventral, and dorsal views of the Rhesus macaque cerebral cortex; dots in grayscale indicate cortical types for cortical areas photographed in the plates of the Atlas of von Bonin and Bailey (1947). Allocortical areas are colored in black; agranular mesocortical areas are colored with the darkest gray; dysgranular mesocortical and eulaminate areas are colored in progressively lighter grays. Koniocortical areas are colored in white. **e**, Grayscale of cortical types in **a**´–**d**´

The areas of the Rhesus macaque neocortex according to von Bonin and Bailey (1947), the plates in which cortical type analysis was performed, and their corresponding cortical types are summarized in Tables 5 and 6. We also show the 58 points on the surface of the cerebral cortex of the Rhesus macaque at which micrographs were taken (Fig. 4A–D) and represent cortical types at each of these points in grayscale (Fig. 4A´–D´). We also represent approximate locations of the other two koniocortical areas (primary auditory and primary somesthetic areas), based on analysis of archival Nissl-stained sections used in previous studies by our group. The distribution of agranular, dysgranular, eulaminate I, eulaminate II, eulaminate III, and koniocortical areas across the cortical quilt of the Rhesus macaque follows two gradients of laminar elaboration, paraolfactory and parahippocampal, previously described in this species (Barbas and Pandya 1987, 1989; Pandya et al. 1988, 2015; Barbas and García-Cabezas 2015; Pandya et al. 2015; Hilgetag et al. 2016; García-Cabezas and Barbas 2017; García-Cabezas et al. 2019). Cortical type maps (Fig. 4A´–D´) show that isocortical (eulaminate) areas account for about 75% of all cortical areas and cover more cortical surface of the Rhesus macaque than mesocortical (agranular and dysgranular) areas. Of note, all agranular areas are in continuity forming a closed ring at the limit of the hemisphere. All dysgranular areas also form a continuum, and so do eulaminate I, and eulaminate II areas. In contrast, eulaminate III areas (1) in the DLPFC; (2) in frontal motor, anterior parietal, and middle part of the temporal lobe; and (3) in the occipital lobe are surrounded by eulaminate II areas. The three koniocortical areas are also each surrounded by eulaminate III areas.

**Table 5:** Frontal (F) and parietal (P) areas in the cerebral cortex of the Rhesus macaque according to the Atlas of von Bonin and Bailey (1947) and their corresponding types

Plate in the Atlas	Abbreviation	Cortical type
Plate I	*FA*	Eu III
Plate II	*FB*	Eu II
Plate III	*FB*	Eu I
Plate IV	*FBA*	Eu III
Plate XXIV	*FC*	Eu I
Plate XXV	*FCBm*	Eu I
Plate XXVI	*FCBm*	Dys
Plate XXVII	*FCop*	Eu II
Plate X	*FD*	Eu I
Plate XVI	*FD*	Eu I
Plate XVII	*FD*	Eu II
Plate XVIII	*FD*	Eu I
Plate XIII	*FD*	Eu III
Plate XX	*FDC*	Dys
Plate XIV	*FDE*	Eu I
Plate XV	*FDE*	Eu I
Plate XIX	*FDL*	Dys
Plate XI	*FD∆*	Eu II
Plate XII	*FDΓ*	Eu III
Plate XXI	*FF*	Dys
Plate XXIII	*FG*	Dys
Plate XXII	*FL*	Dys
Plate IX	*PB*	Eu III
Plate XXVIII	*PCop*	Eu II
Plate XXIX	*PCop*	Eu III
Plate XXXII	*PE*	Eu I
Plate XXX	*PE*	Eu III
Plate XXXI	*PEm*	Eu III
Plate XXXV	*PF*	Eu II
Plate LXII	*PF*	Eu II
Plate XXXVI	*PFC*	Eu II
Plate XXXIV	*PG*	Eu II

**Table 6:** Temporal (T), occipital (O), cingulate (L), and insular (I) areas in the cerebral cortex of the Rhesus macaque according to the Atlas of von Bonin and Bailey (1947) and their corresponding types

Plate in the Atlas	Abbreviation	Cortical type
Plate XXXVIII	*TA*	Eu I
Plate XXXIX	*TA*	Eu I
Plate XL	*TB*	Eu II
Plate VIII	*TC*	Eu I
Plate XXXVII	*TCB*	Eu I
Plate LI	*TE*	Eu II
Plate LII	*TE*	Eu II
Plate LIII	*TE*	Eu II
Plate L	*TEO*	Eu II
Plate LIV	*TF*	Dys
Plate LVI	*TF*	Eu II
Plate XLI	*TG*	Dys
Plate XLII	*TG*	Dys
Plate LV	*TH*	Dys
Plate XLV	*OA*	Eu III
Plate XLVI	*OA*	Eu III
Plate XLVII	*OA*	Eu II
Plate XLVIII	*OB*	Eu II
Plate XLIX	*OB*	Eu III
Plate VII	*OC*	Konio
Plate V	*LA*	Ag
Plate VI	*LA*	Ag
Plate XXXIII	*LC*	Dys
Plate LVII	*LC*	Eu I
Plate XLIV	*IA*	Ag
Plate XLIII	*IB* (not shown in the map)	Dys

### Areas in the human ventromedial prefrontal cortex (VMPFC) are mesocortical and isocortical

**Fig. 5:**
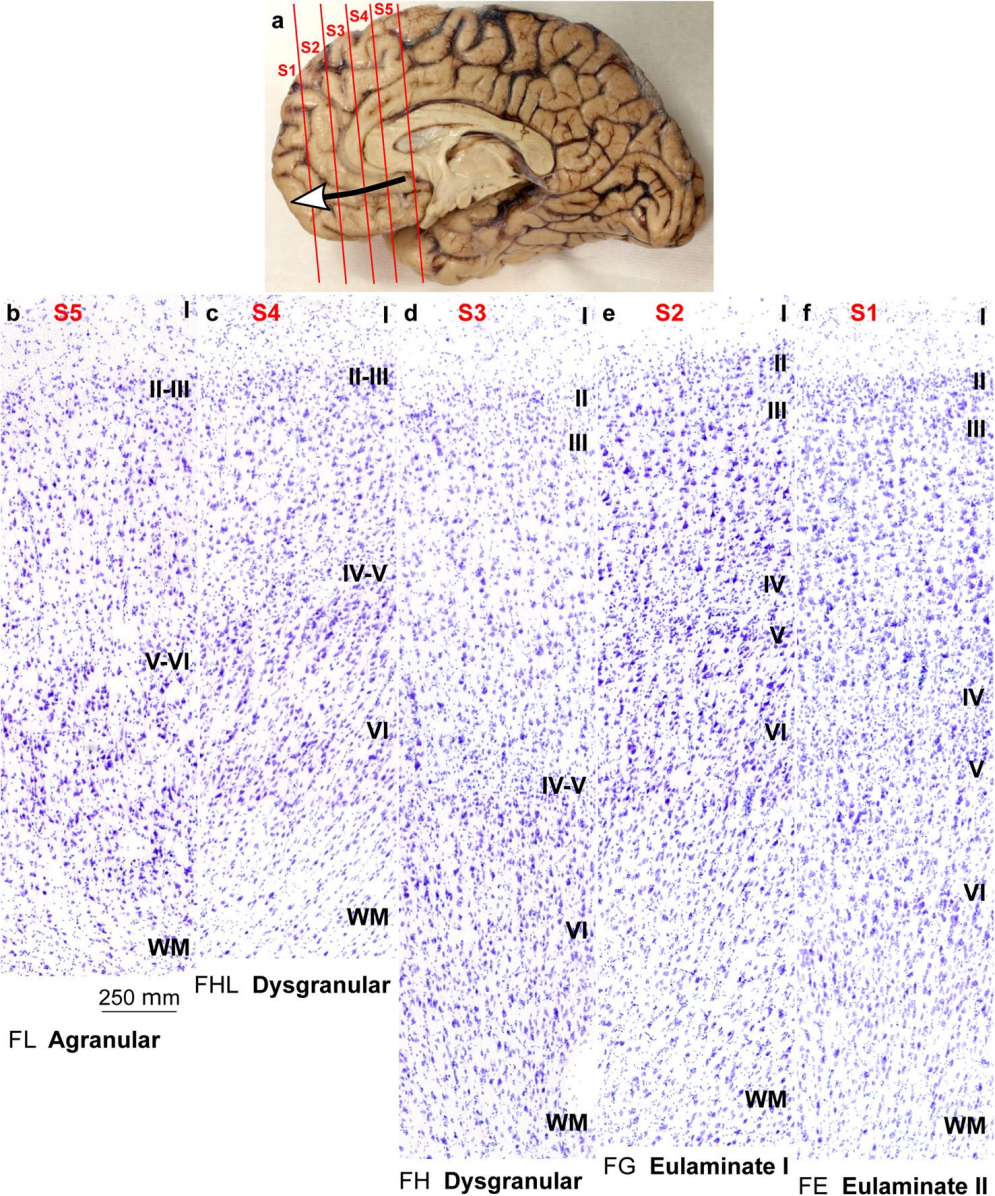
Cortical types across the human ventromedial prefrontal cortex. **a**, View of the left brain hemisphere (case HCD); red lines indicate planes of separation of coronal slabs; black and white arrow indicates gradient of laminar differentiation in the ventromedial prefrontal cortex (VMPFC). **b–f**, Micrographs of areas across the VMPFC (Nissl staining) at the levels indicated in A. Cortical areas, according to von Economo and Koskinas (1925/2008), and cortical types, according to laminar features of Table 3, are indicated below each micrograph. S1, S2, S3, S4, S5, indicate coronal slabs from anterior to posterior. Roman numerals indicate cortical layers. WM: white matter. Calibration bar in b applies to b–f

We have charted cortical types across the human cortex in a previous article (García-Cabezas et al. 2020). Here, we analyzed cortical types and laminar gradations across the human VMPFC (Fig. 5A) according to laminar features of Table 3 to provide direct comparison of cortical gradients of laminar elaboration between primate (human) and rodent (rat) species. These gradients are described in the VMPFC of humans spanning from the allocortical precommissural part of the hippocampal formation (taenia tecta) to eulaminate areas in the frontal pole (Mackey and Petrides 2014; García-Cabezas et al. 2020).

The areas in the VMPFC adjacent to the precommissural part of the hippocampal formation (taenia tecta) had overall simple laminar differentiation and lacked layer IV. Deep layers (V–VI) in these areas had more neurons densely packed than superficial layers (II–III) that were sparsely populated. The largest bodies of pyramidal neurons were in layer V and layers I and II were separated by irregular boundary (Fig. 5B). We categorized these areas as *Agranular*.

More anterior areas in the VMPFC had rudimentary layer IV. In these areas, deep layers (V–VI) were slightly more prominent than superficial layers (II–III), the largest pyramidal neurons were in layer V, and layers I and II were separated by slightly irregular boundary (Fig. 5C, D). We categorized these areas as *Dysgranular*.

Other areas even further anterior in the VMPFC had thin but continuous layer IV with deep layers (V–VI) as prominent as superficial layers (II–III). The largest bodies of pyramidal neurons were equally distributed in layers III and V, layers V and VI were more distinct than in agranular and dysgranular areas and the border between layers I and II was sharper (Fig. 5E). We categorized these areas as *Eulaminate I*.

Finally, frontal pole areas had thicker layer IV than Eulaminate I areas, superficial layers (II–III) were more prominent than deep layers (V–VI), and the largest bodies of pyramidal neurons were in layer III. Layers V and VI were distinct layers with sublayers and the border between layers I and II was also sharp (Fig. 5F). We categorized these areas as *Eulaminate II*.

### Eulaminate III areas in primates are not contiguous and surround koniocortical areas

In rats, eulaminate areas form an island surrounded by dysgranular areas (Fig. 3). In Rhesus macaques and in humans the island of isocortex surrounded by mesocortex shows several types of progressively more complex laminar elaboration (Figs. 4, 5). Cortical areas of eulaminate I type form a continuum surrounded by dysgranular areas. Eulaminate II areas form yet another continuum surrounded by eulaminate I areas. In contrast, eulaminate III areas are not adjacent to each other, do not form a continuum, and are surrounded by eulaminate II areas. Similarly, koniocortical areas are not contiguous with each other and are surrounded by eulaminate III areas (see primary sensory areas in Fig. 4).

**Fig. 6:**
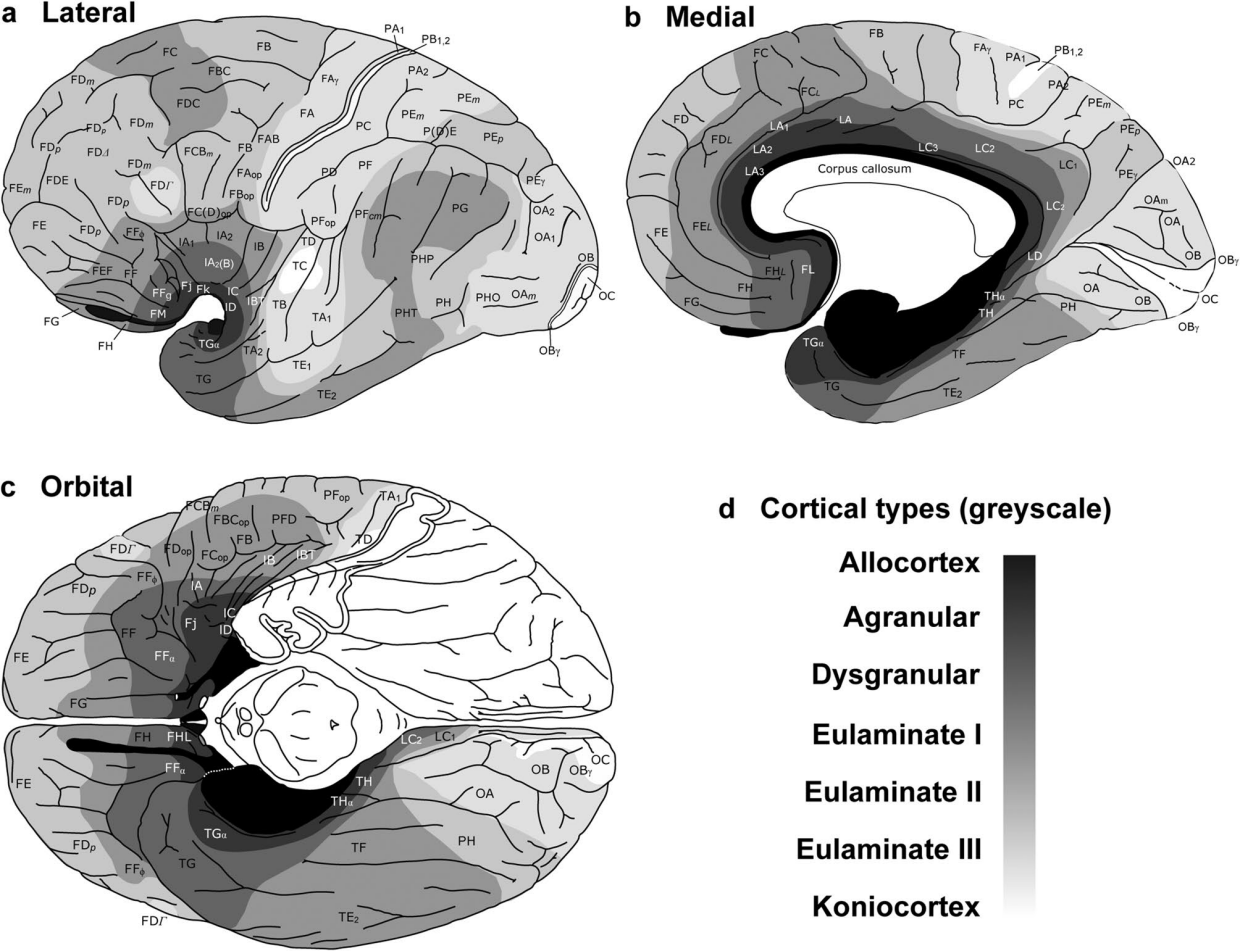
Distribution of cortical types across the human cortex. Lateral (**a**), medial (**b**), and orbital (**c**), views of the human brain. Cortical areas are indicated according to von Economo and Koskinas (1925/2008); cortical types are colored in grayscale according to García-Cabezas et al. (2020). Allocortical areas are colored in black; agranular mesocortical areas are colored with the darkest gray; dysgranular mesocortical and eulaminate areas are colored in progressively lighter grays. Koniocortical areas are colored in white. **d**, Grayscale of cortical types in **a**–**c**. This figure is modified from a previous article of our group [see Fig. 8 from García-Cabezas et al. (2020)] published under Creative Commons CC-BY license that grants to third parties all content of the article (https://www.frontiersin.org/legal/copyright-statement)

We reproduce in Fig. 6 the map of cortical types of the human cortex of our previous article (García-Cabezas et al. 2020) to highlight that, like in the Rhesus macaque, eulaminate III and koniocortical areas are discontinuous. The gradient of laminar elaboration across the human VMPFC continues dorsally into the dorsolateral prefrontal cortex (DLPFC) and reaches eulaminate III areas; these areas have the most complex laminar elaboration in the DLPFC of humans and primates and are entirely surrounded by eulaminate II areas (Figs. 4, 6). In the human neocortex, there are three islands of eulaminate III cortex: (1) in the DLPFC; (2) in frontal motor, anterior parietal, and middle part of the temporal lobe; and (3) in the occipital lobe [Fig. 6; García-Cabezas et al. (2020)]. The three islands of eulaminate III type cortex in humans are not adjacent with each other and they are entirely surrounded by eulaminate II areas. Also, the three koniocortical areas of the primate cerebral cortex are surrounded by eulaminate III areas in the parietal (primary somesthetic area), temporal (primary auditory area), and occipital (primary visual area) lobes [Figs. 4, 6; García-Cabezas et al. (2020)].

### Gradients of laminar elaboration expand in primates by the addition of eulaminate types

**Fig. 7:**
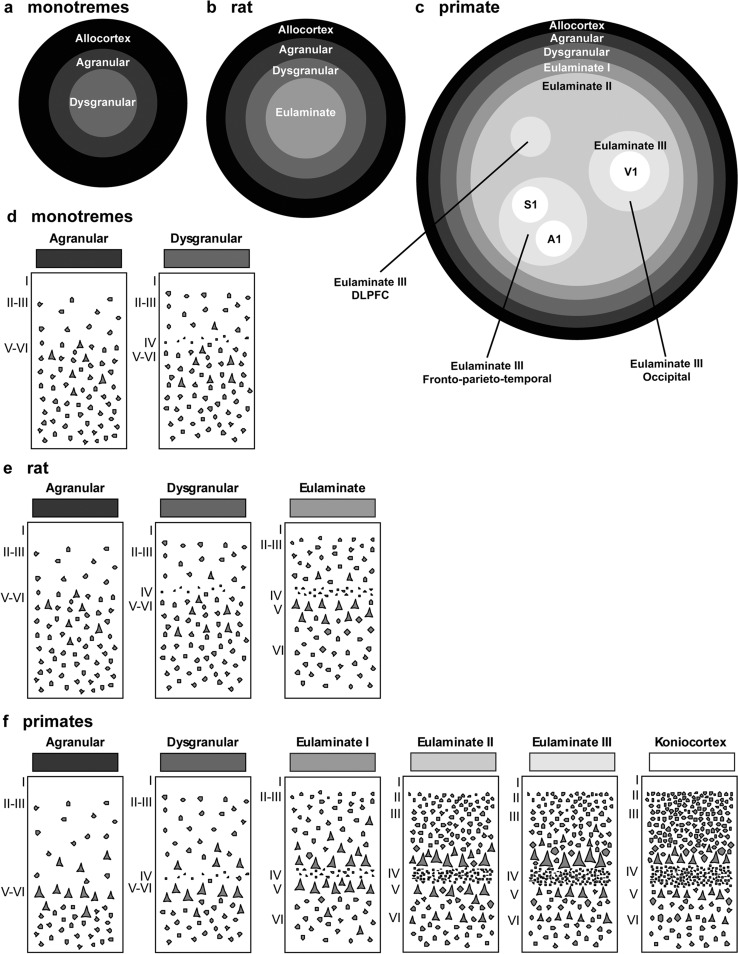
Distribution of cortical types in simplified flat maps of monotremes, rats, and primates. **a–c**, Simplified flat maps of the cerebral cortex of monotremes (**a**), rats (**b**), and primates (**c**). Cortical types are colored in grayscale; allocortical areas are colored in black; agranular mesocortical areas are colored with the darkest gray; dysgranular mesocortical and eulaminate areas are colored in progressively lighter grays. Koniocortical areas are colored in white. **d**, Cartoons of types of neocortical areas in monotremes. **e**, Cartoons of types of neocortical areas in rats. **f**, Cartoons of types of neocortical areas in primates. Roman numerals indicate cortical layers. *A1* primary auditory area, *DLPFC* dorsolateral prefrontal cortex, *S1* primary somesthetic area, *V1* primary visual area

We represented cortical types in simplified flat maps of the cerebral cortex of rats and primates to visualize their topological arrangement across these species. We also represent a map of cortical types in monotremes based on the studies of Abbie (1940) because they are considered the most primitive of all the extant mammals (Deakin et al. 2012). Cortical types are colored in grayscale on target-like flat maps (Fig. 7).

In monotremes, a central island of dysgranular mesocortex is surrounded by agranular mesocortex. The outermost ring is the allocortex (Fig. 7A).

In rats, there is a central island of eulaminate cortex surrounded by rings of dysgranular mesocortex, agranular mesocortex, and the outermost ring of allocortex (Fig. 7B). The eulaminate island is composed of somesthetic and motor areas and some visual areas (see Table 4).

In primates, allocortex, neocortex *non *sensu stricto (agranular and dysgranular mesocortex), and two eulaminate rings (eulaminate I and II) form closed concentric rings. In contrast, eulaminate III areas form three non-connected islands (DLPFC, fronto-parieto-temporal, and occipital). Koniocortical areas (V1, primary visual; S1, primary somesthetic; and A1, primary auditory areas) form three islands surrounded by eulaminate III areas (Fig. 7C). Figure 7D shows cartoons of the two neocortical types identified in monotremes, Fig. 7E shows cartoons of the three neocortical types identified in rats, and Fig. 7F shows cartoons of the six neocortical types identified in primates.

## Discussion

In the present article, we perform cortical type analysis of the entire neocortex of adult rats and Rhesus macaques and provide tables with cortical areas and their corresponding cortical type in both species. We also trace cortical types across the human VMPFC to provide direct comparison of cortical types between rat and human species and represent the distribution of cortical types on maps to visualize topological relations between types in the three species. The main findings of this article are that (1) most of the neocortex of rats is composed of mesocortical areas, while isocortical areas are significantly expanded in Rhesus macaques and humans; (2) mesocortical areas across rat and primate species have comparable laminar architecture; (3) the expansion of the isocortex in primates consists of eulaminate areas of more complex laminar architecture some of which lack counterpart in rats; (4) cortical types are topologically arranged in concentric rings in rats and primates; in both species, eulaminate areas are surrounded by mesocortical areas; (5) in primates, the innermost eulaminate III rings form isolated islands surrounded by the continuous ring of eulaminate II cortex; koniocortical areas also form isolated islands surrounded by rings of eulaminate III cortex.

Below, we will discuss the evolutionary implications of these findings and how they underlie hypotheses on homology of cortical parts between rats and primates. We will address the strengths and potential shortcomings of the Hypothesis on the Dual Origin of the Neocortex and will take into account modern studies of pallial patterning to update the original formulation of this hypothesis. We also propose further experiments to fill the gaps in our explanation of the expansion of the isocortex in primates. Finally, we address the functional implications of the expansion of isocortical areas for cortical processing in primates versus rodents. But first, we will briefly comment some alternatives to cortical type analysis that may be easier to perform for non-experts in neuroanatomy.

### Proxies for cortical types

Cortical type analysis requires assessing laminar features, summarized in Table 3, of a given cortical area under microscopic examination of Nissl-stained tissue. This analysis may be difficult to perform for non-experts in neuroanatomy. Alternatively, estimation of some quantitative features of cortical structure may be used to obtain cortical type-like categorizations in a more automated way.

For instance, the antibody SMI-32 labels a non-phosphorylated intermediate neurofilament protein that is expressed in the neuron body and proximal dendrites of a subset of pyramidal projection neurons in the cortex of macaques and humans. SMI-32-labeled neurons are in layers III and V and, to a lesser extent, in layers II and VI (Campbell and Morrison 1989). Interestingly, the density of SMI-32 neurons positively correlates with cortical type in macaques and humans (García-Cabezas et al. 2020; John et al. 2021). Neuron density, as showed in previous studies (Dombrowski et al. 2001; Hilgetag et al. 2016), also correlates with cortical type. The size of pyramidal neurons in layers III and V measured in micrographs of Nissl-stained sections may also be a good proxy for cortical type analysis (García-Cabezas et al. 2020). Finally, density of intracortical myelin in brain sections stained for myelin or by in vivo estimation using T1w/T2w signal also correlates with cortical type (García-Cabezas et al. 2020; John et al. 2021).

### Cortical types: key to homology hypotheses across rodents and primates

The laminar structure of the neocortex in rats, Rhesus macaques, and humans varies systematically across gradients of laminar elaboration. In the three species, those gradients start adjacent to allocortical areas and progress through mesocortical (neocortex *non *sensu stricto) into isocortical (neocortex sensu stricto) areas. Mesocortical areas show comparable laminar features in rats (Fig. 2A–J) and humans (Fig. 5B–D), but there are significant differences in the laminar architecture of the isocortex of these species. The isocortex in rats represents a small portion of the entire neocortex (Fig. 3) and is composed of areas of the single eulaminate type identified in this species (Fig. 2K–O). In contrast, the isocortex of Rhesus macaques (Fig. 4) and humans (Fig. 6) expands significantly with the addition of cortical types of progressively more complex laminar elaboration (e.g., Fig. 5E, F). This expansion is better visualized in simplified flat maps of the cerebral cortex of monotremes, rats, and primates (Fig. 7). Thus, the expansion of the isocortex in primates is both quantitative and qualitative with the emergence of eulaminate types that are not identified in rats.

A key feature of cortical types in rats and primates is their topological arrangement: allocortex, mesocortex (neocortex *non *sensu stricto), and isocortex (neocortex sensu stricto) are arranged in concentrical rings with the same topological ordering across these species (Fig. 7). The outermost cortical ring in rats, Rhesus macaques, and humans is composed of olfactory and hippocampal allocortical areas [including the singular entorhinal schizocortex; Puelles et al. (2019)]. In the three species, the allocortex surrounds the agranular mesocortical ring which surrounds the dysgranular mesocortex. Finally, the isocortex of rats and primates forms an island surrounded by mesocortical and allocortical rings. In primates, this topological arrangement emerges in development (Barbas and García-Cabezas 2016; García-Cabezas et al. 2019) suggesting that the most eulaminate areas of the primate isocortex (eulaminate II, eulaminate III, and koniocortex) likely emerged in evolution as progressive expansion of the gradients of laminar elaboration in an ancestor common to rodents and primates.

Therefore, according to the Hypothesis on the Dual Origin of the Neocortex, the allocortex and the outer rings of the primate neocortex (agranular and dysgranular mesocortical areas) are likely homologues with the equivalents in the rat counterparts because they show comparable laminar structure (Fig. 2A–J; Fig. 5B–D) and comparable topological arrangement across the cortical quilt of these species. In contrast, eulaminate areas with the simplest laminar architecture in primates are likely homologous with areas in the island of isocortex of rats, but highly eulaminate neocortical areas of the innermost rings of the primate cortex do not seem to have homologous with areas in the rat (Fig. 7). These homology hypotheses are supported by developmental studies of laminar gradients that show (1) comparable architecture of prospective mesocortical areas in humans with overall neocortical areas in rats and (2) progressive development across human prospective eulaminate areas of the OSVZ, a primate specialization that is not identified in rats (Barbas and García-Cabezas 2016; García-Cabezas et al. 2019).

Some evolutionary biologists distinguish homologue (biological) characters and homologue states (Wagner 2014). According to them, an evolutionary transformation series (in the present case, the expansion of cortical gradients of laminar elaboration) is a homologous character; in contrast, homologous character states comprised by the transformation series (in the present case, different degrees of expansion of cortical gradients of laminar elaboration) represent this homologue character in two or more species. Thus, the gradient of laminar elaboration of the cerebral cortex could be considered a biological character; gradients with fewer or more types (like those represented in the flat maps of Fig. 7) could be considered character states.

### Expansion of cortical gradients: differences between macaques and humans

Is there any difference in the expansion of gradients of laminar elaboration between macaques and humans? Are cortical types identical in these species? The laminar features summarized in Table 3 allow defining equivalent and comparable cortical types in macaques and humans, suggesting that comparable qualitative cortical types can be defined across primate species. Nevertheless, this does not imply that homologous types across primate species will have the same cellular and connectional features; for instance, glia/neuron ratio (Sherwood et al. 2006), gray/cell index (Casanova et al. 2009), and dendritic arborization of pyramidal neurons (Elston et al. 2011) vary across the cortex of primate species. We hypothesize that these features will vary systematically across the template of cortical types in each primate species. Regarding the expansion of gradients, a close inspection of Fig. 4 (cortical types on the surface of the macaque cortex) and Fig. 6 (cortical types on the surface of the human cortex) shows that the relative extension of limbic types across the cortical surface is preserved in macaques and humans; in contrast, the extension of cortical surface covered by eulaminate types is expanded in the human cortex compared to the macaque cortex. Thus, it seems that equivalent cortical types are found across gradients of laminar elaboration in the cortex of primates, but the relative proportion of cortical surface covered by eulaminate (I, II, and III) types increases in humans compared to macaques. Further quantitative studies are needed to confirm this qualitative observation.

### Gaps in the explanation of cortical phylogeny by the Hypothesis on the Dual Origin of the Neocortex

The Hypothesis on the Dual Origin of the Neocortex proposed by Raymond Dart (1934), Arthur A. Abbie (1940, 1942), and Friedrich Sanides (1962, 1970) provides phylogenetical explanations for the tangential expansion of the primate cortex based on cortical type analysis (laminar elaboration) and topology of cortical types across species as studied in adult brains. Some aspects of this hypothesis are compatible with modern developmental data, but others will have to be confirmed or rejected with genoarchitectonic studies of cortical patterning and causal mechanisms of development, which are considered fundamental in contemporary biology for proposing evolutionary hypotheses (Puelles and Medina 2002; Hall 2003).

Modern genoarchitectonic studies show that sectors of the neural tube, like pallial sectors, are specified in primary events of patterning induced by organizers. These organizers secrete morphogenetic proteins that diffuse and form gradients around them. Most morphogenetic proteins modify not instructively gene expression in developing cells in function of positional genomic readouts of their concentration within a gradient. The responding neuroepithelial cells are induced to commit themselves to one among predetermined fates (Echevarria et al. 2003; Puelles and Ferran 2012). Primary events of patterning early in development result in the specification of radial histogenetic domains encompassing the full ventriculo-pial radial dimension of the neural wall (García-Calero and Puelles 2020). Such domains, characterized by distinct molecular profiles demonstrated by genoarchitectonic techniques, are considered fundamental morphological units (FMUs) of the bauplan of the neural tube (Nieuwenhuys and Puelles 2016).

**Fig. 8:**
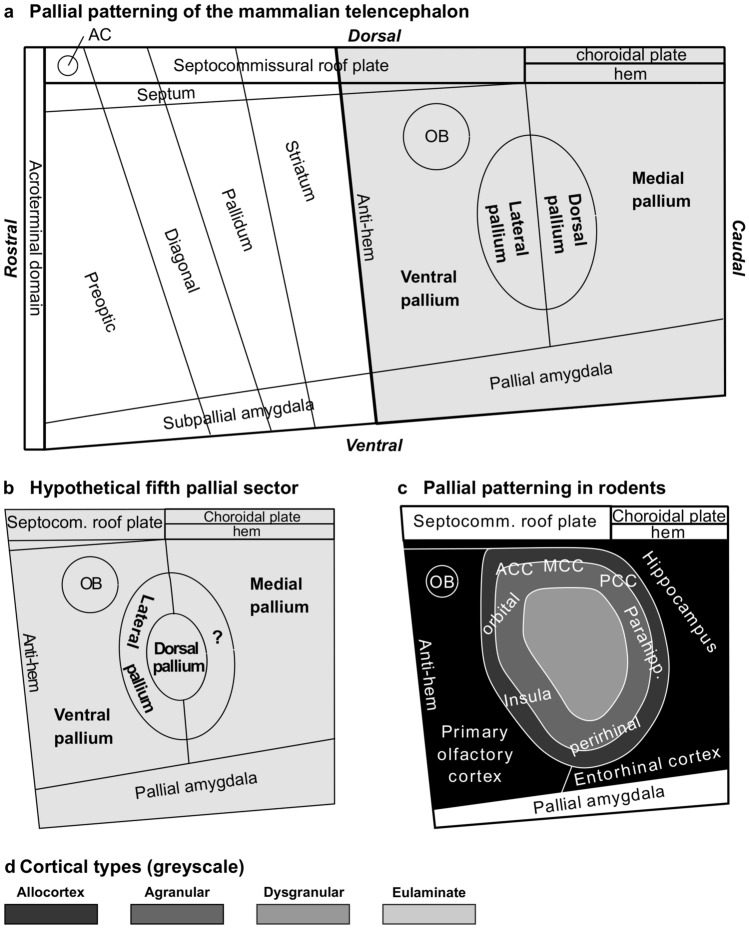
Pallial patterning in mammals. **a**, Sketch of Fundamental Morphological Units (FMUs) of the mammalian telencephalon according to the Prosomeric Model (Puelles and Rubenstein 1993, 2015; Nieuwenhuys and Puelles 2016; Puelles 2018; Puelles et al. 2019). Gray shading indicates pallial FMUs according to the Tetrapartite Model of Pallial Development and Evolution (Puelles 2017, 2021). **b**, Sketch of FMUs of the mammalian pallium showing a hypothetical fifth pallial sector indicated with interrogation sign (?). **c**, Sketch of the rat pallium according to pattering studies in rodents (Puelles et al. 2019); prospective allocortical areas are colored in black; prospective agranular mesocortical areas are colored with the darkest gray; prospective dysgranular mesocortical and prospective eulaminate areas are colored in progressively lighter grays. **d**, Grayscale of cortical types in **c**. Abbreviations: AC, anterior commissure; ACC, anterior cingulate cortex; MCC, middle cingulate cortex; OB, olfactory bulb; PCC, posterior cingulate cortex

The cerebral cortex of vertebrate species seems to develop out of four FMUs (medial, dorsal, lateral, and ventral pallia; Fig. 8A) which are patterned early in development in fish, amphibia, sauropsidian, and mammalian embryos. Fate studies in these species show that the hippocampal formation develops from the medial pallium; the primary olfactory cortex develops from the ventral pallium; mesocortical areas of the insula and the perirhinal cortex develop from the lateral pallium; and most of the neocortex develops from the dorsal pallium. Thus, the allocortex develops across vertebrates from two well-defined FMUs (medial and ventral pallia) and the neocortex (sensu stricto and *non *sensu stricto) from other two FMUs (dorsal and lateral pallia) (Medina et al. 2011; Puelles 2017; Suryanarayana et al. 2017; Pessoa et al. 2019; Puelles et al. 2019). The origin of the cerebral cortex across vertebrates in four pallial sectors has been systematized as the Tetrapartite Model of Pallial Development and Evolution by Luis Puelles (Puelles 2017, 2021). This model has relevant implications for the Hypothesis on the Dual Origin of the Neocortex because it shows that, contrary to what Sanides and his contemporaries believed [e.g., Filimonoff (1947)], the rings of mammalian neocortex did not appear de novo in mammals out of rudimentary anlagen identified in reptiles but expanded within pallial sectors (lateral and dorsal pallia) that were likely present in the common ancestor of fish and land vertebrates.

### Updates in the dual origin of the neocortex hypothesis

After specification, several secondary events of histogenesis (e.g., neurogenesis, cell migration, axon navigation, cytoplasmic differentiation, synapse formation, myelination, synapse pruning…) take place in FMUs. The time-course and intensity of secondary events, like neurogenesis, varies across FMUs and results in the final morphogenesis (tertiary events like formation of grisea—nuclei, layers) of the brain (Nieuwenhuys and Puelles 2016). Thus, the distribution of neurons and glial cells in layers and the gradients of laminar elaboration identified in the cerebral cortex of adult brains by microscopic examination of Nissl-stained sections are tertiary events.

One of the limitations of the Hypothesis on the Dual Origin of the Neocortex as proposed by Sanides is that it is based on tertiary events, but still provides hints on the primary events of pallial specification early in development: gradients of laminar elaboration in rats and primates (Barbas and García-Cabezas 2016; García-Cabezas et al. 2019) may have resulted from gradients of morphogenetic signals during pallial patterning; such gradients may have acted longer in primates compared to rodents producing eulaminate II, eulaminate III, and koniocortical rings in primates. In this sense, several organizers have been described or theoretically proposed to explain patterning of the cerebral cortex [see Puelles et al. (2019)], but we still lack fundamental data to get a full picture of pallial specification.

Here, we propose and updated version of the Dual Origin of the Neocortex Hypothesis (Box 1). This updated version complements the insights of Sanides in cortical phylogenesis with modern knowledge on causal mechanisms of cortical patterning (Puelles et al. 2000, 2019; Medina et al. 2011; Puelles 2017; Suryanarayana et al. 2017; Pessoa et al. 2019). We frame the updated version of the Dual Origin of the Neocortex Hypothesis within two theories/models that explain the development of the neural tube [the Prosomeric Model (Puelles and Rubenstein 1993, 2015; Nieuwenhuys and Puelles 2016; Puelles 2018); see Fig. 8A] and of the cerebral cortex [The Tetrapartite Model of Pallial Development and Evolution (Puelles 2017, 2021); see Fig. 8A]. The updated version of the Hypothesis of Sanides also has implications for understanding cortical hierarchies explained by the Structural Model (Barbas 1986; Barbas and Rempel-Clower 1997; García-Cabezas et al. 2019) as outlined in the last heading of this article.

The updated Hypothesis on the Dual Origin of the Neocortex leaves many open questions to be addressed experimentally in the future. The most relevant one is discovering the origin of several mesocortical areas, like cingulate areas. It is known that allocortical areas originate in medial and ventral pallia, insular and perirhinal mesocortical areas originate in the lateral pallium, and the rest of neocortical areas originate in the dorsal pallium (Puelles et al. 2019). Cortical type analysis of adult rats and primates in the present article suggest the existence of another pallial sector that would originate cingulate mesocortical areas. Such hypothetical sector would be rostral to the medial pallium and would form a close ring with the lateral pallium encircling the dorsal pallium (Fig. 8B) which would explain the topological relations of cortical types (Fig. 8C). This hypothetical pallial sector has been suggested independently by our group (García-Cabezas et al. 2019) and by Puelles and his coworkers (Puelles et al. 2019). Future studies of pallial patterning in mammals and other vertebrate species will have to address the existence of a fifth pallial sector, as well as the sources of morphogenetic signals that pattern the ring of mesocortex between allocortex and isocortex.

**Fig. 9:**
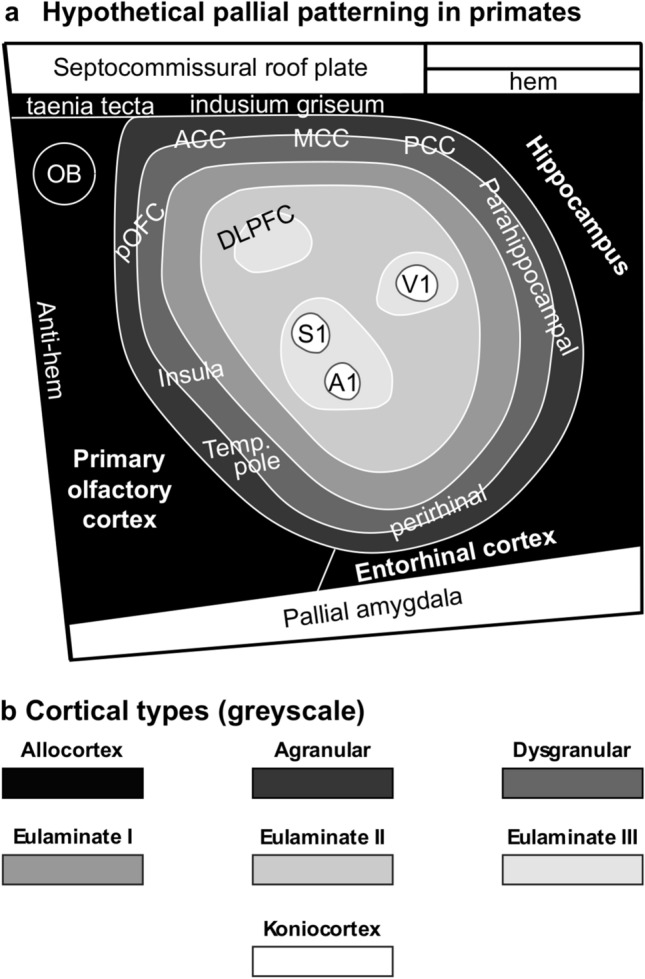
Pallial patterning in primates. **a** Sketch of hypothetical pallial patterning in primates; agranular mesocortical areas are colored with the darkest gray; dysgranular mesocortical and eulaminate areas are colored in progressively lighter grays. Koniocortical areas are colored in white. **b** Grayscale of cortical types in **a**. *A1* primary auditory area, *ACC* anterior cingulate cortex, *DLPFC* dorsolateral prefrontal cortex, *MCC* middle cingulate cortex, *OB* olfactory bulb, *PCC* posterior cingulate cortex, *pOFC* posterior orbitofrontal cortex, *S1* primary somesthetic area, *Temp. pole* temporal pole, *V1* primary visual area

Future developmental studies in primates will also have to address the sources of morphogenetic signals that pattern the non-adjacent islands of eulaminate III isocortex and of primary sensory koniocortical areas (Fig. 9). We also lack information about the time-course of primary events of pallial specification: Are allocortical and mesocortical rings patterned initially together with an undifferentiated island of isocortex? Is there a second phase of patterning for eulaminate rings in primates? There is a large field of discoveries in pallial patterning of primates waiting to be uncovered by developmental neurobiologists in the coming decades.

Box 1. Updated Hypothesis on the Dual Origin of the Neocortex

**Table:** 

Principles of the Tetrapartite Model of Pallial Development and Evolution
1. The cerebral cortex originates from four Fundamental Morphological Units (FMUs) specified in the alar plate of the peduncular hypothalamic prosomere
2. The four FMUs that originate the cerebral cortex (medial, ventral, lateral, and dorsal pallia) are identified across fish, amphibia, reptiles, birds, and mammals
3. The olfactory allocortex originates form the ventral pallium and the hippocampal allocortex, including the entorhinal cortex, originates from the medial pallium
4. Mesocortical (neocortex non sensu stricto) and isocortical (neocortex sensu stricto) areas of mammals originate from lateral and dorsal pallia
Principles of the updated hypothesis on the dual origin of the neocortex
5. Allocortical, mesocortical, and isocortical areas in adult brains are topologically arranged in concentric rings across mammals. The allocortex is the outermost ring and the isocortex is the innermost ring. The mesocortical ring lies between the allocortex and the isocortex
6. Neocortical (mesocortical and isocortical) areas show progressive laminar elaboration across gradients that start in periallocortical (agranular mesocortical) areas and progress through proisocortical (dysgranular mesocortical) areas into isocortical (eulaminate) areas
7. Two gradients of laminar elaboration are identified in the cerebral cortex of adult mammals: paraolfactory and parahippocampal. These gradients converge in eulaminate areas
8. Cortical types can be defined operationally across cortical gradients of laminar elaboration
9. Allocortical, mesocortical, and simple isocortical (eulaminate) types are identified in rats and in primates
10. Highly eulaminate types (eulaminate II, eulaminate III, and koniocortical areas) are identified in primates but do not have counterpart in rats
11. The qualitative and quantitative expansion of the primate isocortex emerges in development with progressive development of the outer subventricular zone (OSVZ) in prospective isocortical areas
12. Thus, according to 8, 9, and 10, gradients of laminar elaboration expand quantitatively and qualitatively in primates compared to rodents with the addition of highly eulaminate types
13. Two cortical areas in two mammalian species are likely homologues if they are in the same ring (cortical type) of cortex
14. Mesocortical areas and simple eulaminate areas in rats and primates are likely homologues. Highly eulaminate types (eulaminate II, eulaminate III, and koniocortical areas) in primates do not have homologues in rats
Principles of the structural model for cortical connections
15. Laminar patterns of cortico-cortical connections are related to cortical types
16. Cortical type predicts position of cortical areas across cortical hierarchies

### Primary sensory (koniocortical) areas in primates are evolutionary novelties

The idea that highly eulaminate areas (e.g., koniocortical areas) are evolutionary innovations in primates, contrasts with other hypotheses of neocortical evolution that considers primary sensory and primary motor areas homologous across all mammals. According to this hypothesis, a general plan of neocortical organization has been observed across mammalian species investigated so far, consisting of a constellation of cortical fields that participate in sensory and motor processing: primary visual (V1), somatosensory (S1), auditory (A1), and motor (M1) areas. The proponents of this hypothesis consider that V1, S1, A1, and M1 fields are homologous across mammalian species because they share similar thalamocortical projections, have common architectonic appearance, and their neurons have similar physiological properties. Thus, the primate neocortex would have evolved out of the common ancestor of all mammals by addition and expansion of cortical fields between the allocortex and primary (V1, S1, A1, and M1) areas (Diamond and Hall 1969; Krubitzer and Seelke 2012; Kaas 2019).

The hypothesis of a constellation of primary sensory and motors areas that are homologous across all mammals has several flaws as outlined below. First, as shown in the present article, the laminar architecture of primary sensory areas in rats differs sharply from koniocortical areas in primates. Second, the “sameness” of primary sensory areas across mammals in the constellation of cortical fields hypothesis is based on physiological and functional similarities. We agree with Puelles (2021) in rejecting synaptic connections as proper criteria for supporting homology hypotheses. Having comparable connections and physiological properties makes visual areas in rats and primates analogous, but not homologous. Homology refers to common phylogenetic origin; thus, two body characters, like parts of the neocortex, in two species are homologous if they originated in the same organ of a common ancestor species (Wiley and Lieberman 2011; Wagner 2014). By definition, such statements are hypothetical and indemonstrable, but criteria related to body plans and causal mechanisms in development can be used to propose more substantiated homology hypotheses (Wagner 1989; Puelles and Medina 2002; Puelles 2021). For instance, two characters in two species are likely homologous if they have comparable topological position in the body plan of the two species of interest and if they share comparable causal mechanisms of development in these species. The proponents of the hypothesis of a constellation of primary sensory and motor fields that are homologues across mammals (Diamond and Hall 1969; Krubitzer and Seelke 2012; Kaas 2019) do not take into account the topological relationships of primary sensory areas with other eulaminate areas in rodents and primates. Also, they do not consider that primary sensory areas in primates develop at the end of laminar gradients of elaboration, which suggest sustained and extended action of morphogenetic gradients and organizers in primates compared to rodents.

In contrast to the hypothesis of a constellation of primary sensory and motor fields that are homologues across mammals (Diamond and Hall 1969; Krubitzer and Seelke 2012; Kaas 2019), our updated Hypothesis on the Dual Origin of the Neocortex (Box 1) takes into account topological relations of cortical areas across rings of laminar elaboration in the brains of adult mammals and is not in contradiction with current knowledge on the primary events of specification and causal mechanisms of pallial patterning. Topological relations of cortical types in adult brains preserve the invariant topological neighborhood relations of FMUs specified due to primary events of specification in early development (Nieuwenhuys and Puelles 2016). Thus, the progressive expansion of neocortical gradients of laminar elaboration observed from marsupials and monotremes to rodents and from rodents to primates (Fig. 7) provides a topological framework for homology hypotheses of cortical areas: two areas (e.g., agranular areas in the cingulate) in two species (rats and humans) may be homologous if they are in the same concentric ring (agranular mesocortex), but they are not likely to be homologous if they are in different rings. Therefore, primary sensory (visual, somesthetic, and auditory) areas in rats and primates are likely not homologous, because identical primordia (rings) are not identified in these species. To provisionally accept or reject this homology hypothesis, cortical type analysis in the cerebral cortex of adult prosimians and genoarchitectonic experiments in embryos of these species will be needed. For instance, the primary visual area of tree shrews, prosimians, monkeys, and apes seem to have comparable laminar architecture (Balaram and Kaas 2014), suggesting the emergence of the visual koniocortex in the common ancestor of these species.

### Functional implications of laminar elaboration across the cortex: interspecies differences

Gradients of laminar elaboration described by neuroanatomists across the cerebral cortex of adult humans and other mammals [*e.g.*, Sanides (1970); Mesulam and Mufson (1982); Barbas and Pandya (1989); Hilgetag et al. (2016)] are key for understanding cortical function because they are related to progressive complexity of cortical representations from primary sensory to multimodal areas, described by physiologists [*e.g.*, Hubel and Wiesel (1965)]. Thus, on one extreme of neocortical gradients, cortical areas with the most complex laminar elaboration, like koniocortical (primary sensory) areas, have the simplest sensory representations; in contrast, at the other extreme of neocortical gradients, agranular mesocortical areas with the simplest laminar elaboration have the most complex sensory and mental representations (Mesulam 1985, 1998; Barbas 1995, 2015). Accordingly, physiological elaboration (from koniocortical to agranular mesocortical) counter-parallels laminar elaboration (from agranular mesocortical to koniocortical). This relationship between architecture and physiology is one key to understand the distribution of hierarchies of sensory processing across laminar gradients in the cerebral cortex. The other key, outlined below, is the relation between laminar elaboration and cortico-cortical connections.

Projections ascending along cortical hierarchies are defined as feedforward and projections descending along cortical hierarchies are defined as feedback. Connections between areas at the same level of cortical hierarchies are defined as lateral. Feedback, feedforward, and lateral projections have characteristic laminar patterns of connections. Thus, feedforward projections originate mostly in superficial (II–III) layers and terminate in middle-deep (IV–VI) layers; feedback projections originate mostly in deep (V–VI) layers and target superficial layers (I–III); lateral projections originate in superficial (II–III) and deep (V–VI) layers and terminate across all layers (I–VI) (Rockland and Pandya 1979; Felleman and Van Essen 1991). Cortical types are related to laminar patterns of cortico-cortical connections, and, therefore, to cortical hierarchies, in a relational model called the Structural Model for Cortical Connections (Box 1). According to this model, projections from areas of complex laminar elaboration to areas of simpler laminar elaboration, have “feedforward” laminar patterns. In the reverse direction, pathways from areas of simpler laminar elaboration to areas of more complex laminar elaboration have “feedback” laminar patterns. When two areas of comparable laminar elaboration are connected, projections have “lateral” patterns (Barbas 1986; Barbas and Rempel-Clower 1997; García-Cabezas et al. 2019).

Thus, the topological position of a given cortical area across gradients of laminar elaboration is a predictor of its position across cortical hierarchies. In summary, gradients of laminar elaboration and cortical types described by microscopic examination of Nissl-stained sections (Sanides 1970; García-Cabezas et al. 2020) allow for predictions of laminar patterns of cortico-cortical connections between cortical areas (Barbas 1986; Barbas and Rempel-Clower 1997; García-Cabezas et al. 2019), and for the complexity of sensory representations across cortical areas (Mesulam 1985, 1998; Hilgetag and Goulas 2020; Tucker and Luu 2021). All these principles obtained from studies in human and non-human primates are extrapolatable to the human cerebral cortex and allow for predicting the position of a given cortical area in cortical hierarchies if one knows the cortical type of this area. Such predictions can be done in human subjects in vivo because the content of myelin increases in parallel with laminar elaboration (although there are some exceptions); thus, T1w/T2w maps obtained by MRI can be used as proxies of maps of cortical types (García-Cabezas et al. 2020).

Hilgetag and coworkers define a “natural cortical axis” that aligns cortical hierarchies, gradients of laminar elaboration, and other cellular and molecular features of cortical neurons (Hilgetag and Goulas 2020; Goulas et al. 2021). The updated Hypothesis on the Dual Origin of the Neocortex (Box 1) provides phylogenetic explanation for the expansion of such axis from rodents to primates. Actually, previous studies in mice showed that cortical hierarchies are shallower than in primates (Goulas et al. 2017; Fulcher et al. 2019), a functional feature likely due to laminar gradients less expanded than in primates.

## Conclusions

The topological relations of cortical types defined across gradients of laminar elaboration in mammalian species are at the base of the Hypothesis on the Dual Origin of the Neocortex. This hypothesis takes into account the invariant topological neighborhood relations of cortical types in adult mammals and provides a theoretical framework that allows for proposing homology hypotheses of parts of the cortex across mammalian species. Accordingly, mesocortical areas are homologous across rats and primates, but highly eulaminate areas in primates do not have homologues with the equivalents in the rat counterparts.

Homology hypotheses based on cortical type analysis and the Hypothesis on the Dual Origin of the Neocortex, like those proposed and discussed in the present article, could be further supported with studies of pallial patterning in embryos of rats and primates. Specifically, future developmental studies could search in primate embryos for a hypothetical fifth pallial sector between medial and dorsal pallia and for the sources or mechanisms of morphogenetic signals that pattern the mesocortical ring, the non-connected islands of eulaminate III isocortex, and primary sensory koniocortical areas. Finally, the causal mechanisms underlying the formation of cortico-cortical connections across gradients of laminar elaboration will be a major question to address in future studies.

## Data Availability

All the data used are available in the Tables and Figures of this article.
